# Retesting reverse-reward performance in capuchin monkeys (*Sapajus apella*) after 16 years: evidence of aging-related decline

**DOI:** 10.1007/s10329-026-01246-3

**Published:** 2026-03-09

**Authors:** Yui Sugimoto, James R. Anderson, Hika Kuroshima

**Affiliations:** 1 Graduate School of Letters, Department of Psychology, Kyoto University, Kyoto, Japan; 2 Wildlife Research Center, Kyoto University, Kyoto, Japan

**Keywords:** Aging, Self-control, Reverse-reward, Learning, Memory, Sapajus apella

## Abstract

In reverse-reward contingency tasks, the subject chooses between two potential rewards differing in value. To receive the higher-value reward, the subject must reach toward the lower-value reward; any reach toward the higher-value reward results in receiving the lower-value reward. In studies on animals, the rewards are usually food items differing in quantity or quality. Sixteen years after participating in reverse-reward tests, captive adult capuchin monkeys (*Sapajus apella*) were retested under the same conditions. We asked whether there would be evidence of memory of previous learning, or aging-related effects on performance. As previously, monkeys first experienced a “quantity” condition and then switched to a “quality” condition, or vice-versa. The two best-performing monkeys 16 years earlier showed significantly poorer performance in the present study, indicating possible age-related decline in reverse-reward competence. By contrast, a monkey not yet fully adult 16 years earlier but now 23 years old learned the R-R contingency in the quality condition, which suggests that inhibitory control ability in this species can be expressed at least into their 20s. Other individuals’ performances were similar to those of 16 years ago, some associated with side preferences. Assessments of age-related changes in inhibitory control need to consider task characteristics and behavioral biases. Combining longitudinal and cross-sectional approaches may be optimal for clarifying the development and later decline of cognitive abilities across the lifespan.

## Introduction

Self-control can be defined concisely as foregoing an immediate reward to obtain a higher-value, delayed reward (Rachlin and Green 1972). Beran (2018, p. 14) offers a more elaborate definition: “the ability or capacity to obtain a subjectively more valuable outcome rather than a subjectively less valuable outcome through choosing and then tolerating a longer delay or a greater effort requirement for obtaining that more valuable outcome”. One way in which self-control has been assessed is the reverse-reward (“R-R”) task, first used with nonhuman primates by Boysen and Berntson (1995). In this task, if the subject chooses the inherently more attractive of two simultaneously presented edible options, it receives the lower-value option as a reward, and vice-versa (see Shifferman 2009 for a review of early studies, and Beran 2023 for a recent discussion). Although the R-R task has neither the delay nor the greater (physical) effort requirements specified in Beran’s (2018) definition, the subject must exercise self-restraint by reaching toward the lower- instead of the higher-value reward. In the absence of interventions aimed at enhancing R-R performance, the task (which Beran 2018 considers as a “self-regulatory inhibition” task) is difficult even for species with relatively advanced cognitive abilities, such as chimpanzees and rhesus macaques (Boysen and Berntson 1995; Murray et al. 2005). Capuchin monkeys also struggle with R-R contingencies, despite their impressive cognitive abilities in a variety of other contexts (see Anderson 1996; Fragaszy and Visalberghi 2004). For example, Anderson et al. (2008) tested capuchin monkeys (*Sapajus apella*) aged between 3 and 13 years on two versions of the task. In the “quantity” condition, two quantities of food (one and four pieces of sweet potato) were simultaneously presented on a tray. When the monkey reached for one quantity the experimenter quickly rotated the tray to bring only the alternative quantity within reach (the monkey always took and ate the reward). In the “quality” condition, the same procedure was used with one commercial monkey pellet (lower quality) and one size-equated piece of apple (higher quality) as the stimulus options. All three monkeys tested first in the quality condition performed well (i.e., above chance) whereas no monkey of four tested first in the quantity condition scored above chance. After 10 sessions the two groups switched conditions; however, in the new conditions no monkey scored above chance. These results suggested that (1) qualitative R-R may be easier to master than its quantitative counterpart, and (2) quality and quantity dimensions are processed differently, suggestions consistent with other research findings (e.g., Glady et al. 2012; Padoa-Sioppa and Assad 2006).

Although a literature exists on age-related decline in several executive function tasks that may relate to self-control in several species of non-human primates as well as humans (Lacreuse et al. 2020), to our knowledge no studies have used the R-R procedure. Here, we re-tested the same capuchin monkeys as in Anderson et al. (2008) after a 16-year period during which they participated in numerous perception and cognition studies but no R-R situations. Might their previous experience have any facilitatory effect on performance after such a long delay? Would they show any evidence of remembering the R-R contingency? If so, they might be expected to perform better than on their initial previous exposure. However, as some of the monkeys were now almost 30 years old and possibly experiencing cognitive decline due to aging (see e.g., Bartus et al. 1980; Lacreuse et al. 2020), their performance might be worse than in the original tests 16 years earlier. Capuchin monkeys, which can live to more than 40 years of age (Fragaszy et al. 2004) have recently been proposed as a suitable model for neuropathological studies of Alzheimer’s disease, although reports of cognitive deficits in elderly capuchins remain scarce (Rodriguez et al. 2024). To our knowledge, this is the first study of R-R performance in older monkeys.

## Method

### Subjects

Seven adult tufted capuchin monkeys (*Sapajus apella*), all with experience of the same task 16 years earlier (when two were not yet fully adult; Anderson et al. 2008) participated in the same conditions (Table 1). The monkeys lived in three subgroups in a series of indoor interconnecting cages with water constantly available. They earned part of their daily food (commercial pellets, fruits, vegetables and eggs) as rewards in experiments, with the rest being given in the late afternoon. The monkeys were highly familiar with voluntarily entering a transport box (for reward), transfer to the test room, the cage and apparatus used for testing, and the experimenters (JA in the original study, YS in the present study).

**Table 1:** Details of the monkeys tested in the present and the previous study

ID		Age in years		
	Sex	Previous	Present	Group
Zilla	Female	13.0	29.7	QuanF
Theta	Female	11.1	27.8	QuanF
Pigmon	Male	9.5	26.2	QuanF
Zinnia	Male	6.4	23.1	QuanF
Heiji	Male	13.1	29.8	QualF
Kiki	Female	11.5	28.2	QualF
Zen^a^	Female	3.7	20.4	QualF

QuantF: Quantity First; QualF: Quality First; see the main text for more details

^a^ Zen’s data were excluded from the analysis because she showed no food preference

## Apparatus

**Fig. 1:**
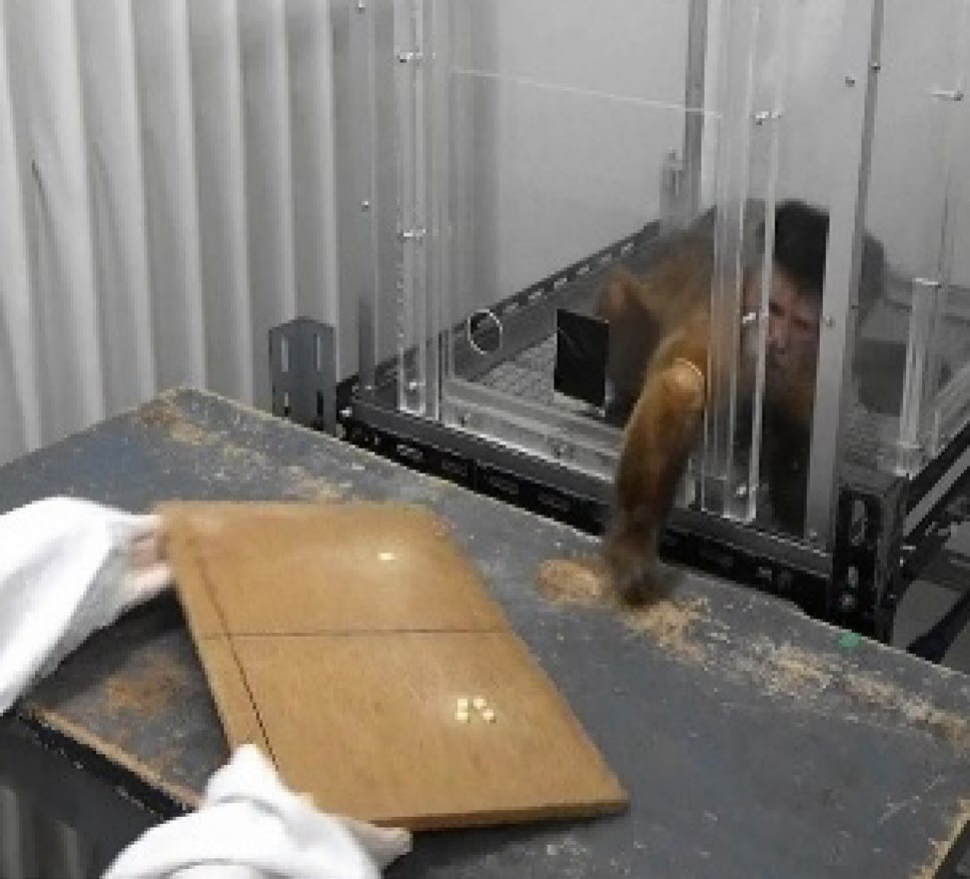
Reverse-reward task. As the subject reaches for four pieces of food, the experimenter turns the tray so the subject can take only the single piece as the reward

The apparatus was that used in the original test. The test cage, 45 cm (W) × 60 cm (D) × 54 cm (H), made of transparent acrylic board with a wire-mesh floor, stood behind a wooden table, 80 cm (W) × 39 cm (D) × 74 cm (H) (Fig. 1). A chair for the experimenter was at the other side of the table, facing the cage. The front panel of the cage had two holes (3.5 cm in diameter) arranged to the left and right of a third, central hole which was sealed with duct tape. The two remaining holes were 16 cm apart and 7 cm above the cage floor, and permitted the monkey to reach unimanually toward items on the presentation tray, which was a plain wooden board (36 × 20 cm) which lay on the table. The subject was released into and removed from the test cage by raising and lowering the front panel as required.

All tests were recorded using a digital video camera (Sony, HDR-CX670) fixed to a tripod diagonally to the right of the experimental cage and focused on the monkey.

## Procedure

The procedure was almost identical to that in Anderson et al. (2008). After the monkey was released into the test cage, the experimenter sat at the other side of the table, facing the monkey. Each monkey was assigned to the same group as in Anderson et al. (2008). In Phase 1, the “Quantity First” group (“QuanF”, 2 males, 2 females) were tested on R-R using one piece versus four pieces of sweet potato, a moderately preferred food. The “Quality First” group (“QualF”, 1 male, 2 females) was tested identically but using one small commercial monkey pellet and one similarly sized piece of apple. Food preference tests consisting of 20 presentations of these two food items showed that apple was significantly preferred to pellet in all seven monkeys but two: one (Heiji) showed a significant preference for pellet, and another (Zen) showed no preference and so her data were not analyzed.

To start a trial, while holding the tray below the table surface and so out of view of the monkey, the experimenter placed the food on the tray (1 and 4 pieces of sweet potato for QuanF subjects, 1 pellet and 1 apple piece for QualF subjects). One food array was on the left side of the tray and one on the right, aligned with the holes in the door of the test cage. The experimenter lifted the tray and placed it the table surface, showed it to the monkey for approximately 5 s, and then pushed the front edge of the tray into contact with the test cage. When the monkey reached through a hole, the experimenter immediately rotated the tray 90^0^, taking the reached-for array away from the monkey and bringing the other array within the monkey’s reach. Thus, if a QuanF monkey reached toward four pieces, only one piece was offered as a reward, and vice-versa. The R-R contingency also applied to QualF monkeys. A reach was defined as any extension of fingers through a hole. For monkeys able to extend their arm through a hole, the food was placed further back on the tray to avoid snatching. Right-left locations of food were randomized across trials except that no more than three identical consecutive trials were permitted in a session. Intertrial intervals were approximately 10 s. Phase 1 consisted of ten 20-trial sessions, with one session per day. Phase 2 was identical to Phase 1 except that the groups switched conditions, so that QuanF monkeys now experienced the quality condition, and QualF monkeys were tested on quantity.

## Analysis

One piece of sweet potato in the quantity condition and the pellet (apple piece for Heiji, who now demonstrated pellet preference) in quality condition were designated as the lower-value food, with the alternative option as the higher-value food. The experimenter recorded which food (higher- or lower-value) and which hole (left or right) monkeys chose on each trial. Correct responses (scored as “1”) were 1 piece chosen in quantity trials, and the less-preferred food chosen in quality trials; incorrect responses were scored as 0.

First, a binomial test was conducted for each subject in each phase using the package R (ver. 4. 5. 0) to determine whether the observed proportion of choosing the lower-value food was significantly different from chance. P-values were then adjusted for multiple comparisons using the Bonferroni correction. Second, to test for any age-related effect on reverse-reward performance, we used a Generalized Linear Model (GLM) from the glmer() function in R for each monkey in each phase. The dependent variable, score (correct/incorrect), was modeled using a logit link function with a binomial error distribution. In each model, study year (previous, present) was included as a fixed effect. P-values were obtained using the Anova () function from the car package in R, which applies Type II Wald chi-square test for fixed tests. Results are reported with Wald Chi-square (χ^2^), degrees of freedom and significance levels (p-values). P-values were adjusted for multiple comparisons using the Bonferroni correction.

## Results

**Fig. 2:**
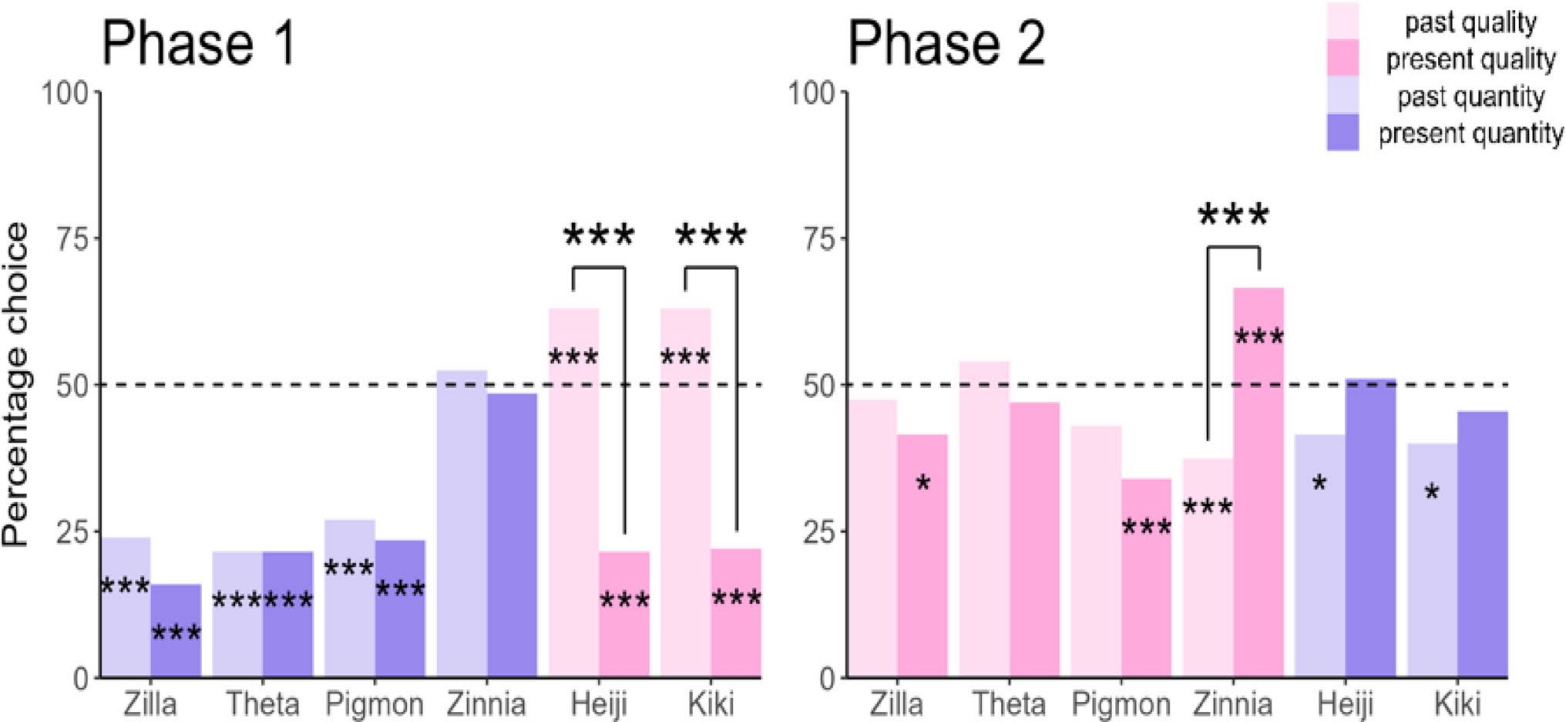
Percentage of choosing the lower-value food item for each year and phase. The left bar of each pair shows performance in the previous study, and the right bar shows performance in the present study. Asterisks in the bars indicate significantly different from chance (binomial test), and those above bars indicate significant differences between years. * *p* < 0.05; *** *p* < 0.001

In the previous study, two monkeys in QualF group (Heiji, Kiki) scored significantly above chance in phase 1; however, in the present study these and the other QualF monkeys all scored significantly below chance (*p* < 0.001). Only one monkey in the QuanF group (Zinnia) scored above chance, doing so in phase 2, after the switch to the quality condition (*p* < 0.001) (see Fig. 2, and supplementary material for full data).

The Type II Wald tests on GLM for individual monkeys in each phase revealed a significant main effect of Year for Heiji-Phase 1 (χ²(1) = 73.08, *p* < 0.001), Kiki-Phase 1 (χ²(1) = 71.14, *p* < 0.001), and Zinnia-Phase 2 (χ²(1) = 34.19, *p* < 0.001). Specifically, the two older monkeys in the QualF group, Heiji (aged 29) and Kiki (aged 28), showed a significant decline in Phase 1 performance compared to previously (present: 21.5% and 22% correct, respectively; previous: 63.0% correct for both). In contrast, Zinnia (aged 23), the youngest subject and in the QuanF group, showed a significant improvement in Phase 2: 66.5% correct compared to 37.5% 16 years earlier. In all the remaining 9 subject-phase combinations no effect of Year was observed, and a strong side-preference was evident in one or both studies (see Table 2).

**Table 2:** Percentages of right-side choice in both studies

ID	Previous study		Present study	
	Phase 1	Phase 2	Phase 1	Phase 2
Zilla	67.0***	85.5***	63.5**	85.5***
Theta	61.5*	94.0***	68.5***	97.0***
Pigmon	59.0	77.0***	63.5**	44.5
Zinnia	54.5	34.5***	11.0***	29.0***
Heiji	51.0	90.5***	62.0*	99.5***
Kiki	38.0*	10.0***	62.0*	93.5***

* *p* < 0.05; ** *p* < 0.01; *** *p* < 0.001

## Discussion

Monkeys that showed successful reverse-reward performance in Phase 1 of the QualF condition 16 years ago failed in the current study. Two plausible reasons come to mind. First, they may simply have forgotten the rule of the R-R task due to the long interval since they had acquired it, with aging-related cognitive decline also contributing. This explanation is consistent with other findings of older capuchin monkeys being more error prone during learning (e.g., Berhane and Gazes 2020). Second, perhaps the monkeys did remember the rule, or even relearned it anew, but were no longer able to effectively execute inhibitory control. For instance, older squirrel monkeys are known to be less capable of inhibiting goal-directed responses compared to younger adults (Lyons et al. 2000). Although diminished visual acuity with age might contribute to the absence of reverse-reward learning, we rule it out here because most monkeys discriminated between food types and food quantities in the preference test and the 1 vs. 4 condition, respectively; inhibitory control, not discrimination was the issue.

One monkey, Zinnia—6 years old (and therefore a subadult) when previously tested and now 23 years old (in prime adulthood)—was the only subject to score significantly above chance on reverse-reward, doing so in Phase 2 (quality condition). It seems likely that cognitive maturation underlies his improved performance. Some other studies suggest that in primates inhibitory control improves into adulthood; for example, Zhou et al. (2016) reported improved anti-saccade performance with age in subadult and young adult rhesus monkeys (4 to 7 years old). However, whereas anti-saccade tasks measure motor inhibitory control, the R-R task requires both motor inhibitory control and reward-based decision-making. Zinnia’s improved performance suggests that the ability to make inhibitory choices and integrate reward value requires cognitive maturation. Another requirement is sufficient rule-learning ability. Future research should distinguish these possibilities by using tasks that separate learning and inhibition.

Two monkeys in the previous study and one in the present study succeeded in the quality condition, whereas no monkey has succeeded in the quantity condition; these outcomes suggest that the latter task is more difficult (also found by Glady et al. 2012), difficult even for some macaques who required over 1,000 trials before learning the R-R contingency (Murray et al. 2005). Our data are also consistent with previous studies showing that capuchins tend to respond differently in quality- and quantity-based decision-making situations (e.g., Drapier et al. 2005; Quintiero et al. 2021; Talbot et al. 2018). However, a particularly notable feature of the data was the appearance of side p, which have been reported in other R-R studies (e.g., Albiach-Serrano et al. 2007; Anderson et al. 2000; Genty et al. 2004; Uher and Call 2008), and which can result in performances near to or at chance level. Cognitive studies on aging should aim to use tasks that are less vulnerable to the emergence of position p.

## Conclusions

Two capuchin monkeys in their late twenties failed a qualitative reverse-reward task on which they had succeeded 16 years earlier, suggesting an age-related decline in inhibitory control. In contrast, one monkey now 23 years old succeeded for the first time, indicating possible strengthening of cognitive ability over a 16-year period from young or sub-adulthood to middle adulthood. Unchanging performances in other monkeys were linked to side preferences. To better understand the trajectory of cognitive control across the lifespan, we recommend combining longitudinal and cross-sectional approaches while taking into account possible confounding variables (e.g., visual acuity).

## Data Availability

The raw data entered into the analyses are available online as Supplementary Information.
