# Publisher Correction: Retesting reverse-reward performance in capuchin monkeys (*Sapajus apella*) after 16 years: evidence of aging-related decline

**DOI:** 10.1007/s10329-026-01257-0

**Published:** 2026-04-15

**Authors:** Yui Sugimoto, James R. Anderson, Hika Kuroshima

**Affiliations:** 1 Graduate School of Letters, Department of Psychology, Kyoto University, Kyoto, Japan; 2 Wildlife Research Center, Kyoto University, Kyoto, Japan

**Correction: Primates**

10.1007/s10329-026-01246-3

In the Abstract of this article, the sentence beginning “Other individuals’ performances were similar...” is incomplete and should have read: “Other individuals’ performances were similar to those of 16 years ago, some associated with side preferences”. This error originated on the publisher’s side, and we deeply regret its occurrence.

The original article has been corrected.

